# Correlation between CT Perfusion Parameters and Microvessel Density and Vascular Endothelial Growth Factor in Adrenal Tumors

**DOI:** 10.1371/journal.pone.0079911

**Published:** 2013-11-18

**Authors:** Hai-yan Qin, Haoran Sun, Xifu Wang, Renju Bai, Yajun Li, Jinkun Zhao

**Affiliations:** 1 Department of Imaging, The Fourth Affiliated Hospital, Harbin Medical University, Harbin, P.R. China; 2 Department of Radiology, The General Hospital, Tianjin Medical University, Tianjin, P.R. China; 3 Department of Radiology, The First Affiliated Hospital, Jiaotong University, Shanghai, P.R. China; 4 Department of Radiology, The Affiliated Cancer Hospital, Tianjin Medical University, Tianjin, P.R. China; University of Central Florida, United States of America

## Abstract

We evaluated the correlation between computed tomography (CT) perfusion parameters and markers of angiogenesis in adrenal adenomas and non-adenomas to determine if perfusion CT can be used to distinguish between them. Thirty-four patients with pathologically-confirmed adrenal tumors (17 adenomas, 17 non-adenomas) received CT perfusion imaging before surgery. CT perfusion parameters (blood flow [BF], blood volume [BV], mean transit time [MTT], and permeability surface area product [PS]) were calculated. Tumor tissue sections were examined with immunohistochemical methods for vascular endothelial growth factor (VEGF) expression and microvessel density (MVD). The mean age of the 34 patients was 43 years. The median BV was significantly higher in adenomas than in non-adenomas [12.3 ml/100 g, inter-quartile range (IQR): 10.4 to 16.5 ml/100 g vs. 8.8 ml/100 g, IQR: 3.3 to 9.4 ml/100 g, *p* = 0.001]. Differences in BF, MTT, and PS parameter values between adenomas and non-adenomas were not significant (*p*>0.05). The mean MVD was significantly higher in adenomas compared to non-adenomas (98.5±28.5 vs. 53.5±27.0, *p*<0.0001). Adenomas also expressed significantly higher median VEGF than non-adenomas (65%, IQR: 50 to 79% vs. 45%, IQR: 35 to 67%, *p* = 0.02). A moderately strong correlation between BF and VEGF (r = 0.53, *p* = 0.03) and between BV and MVD among adenomas (r = 0.57, *p* = 0.02) exist. Morphology, MVD, and VEGF expression in adenomas differ significantly from non-adenomas. Of the CT perfusion parameters examined, both BF and BV correlate with MVD, but only BF correlates with VEGF, and only in adenomas. The significant difference in BV suggests that BV may be used to differentiate adenomas from non-adenomas. However, the small difference in BV shows that it may only be possible to use BV to identify adenomas vs. non-adenomas at extreme BV values.

## Introduction

Adrenal tumors can be malignant or benign, and have high or absent hormone secretion [1], [2]. They comprise approximately 10% of urinary and reproductive system tumors [3], and are found in 3% of autopsies performed in those more than 50 years of age [4]. Incidentally detected adrenal tumors, however, have been reported in 0.35% to 5% of imaging studies performed for other reasons [1], [5]. While the gold standard for diagnosis is the histopathological examination of a tumor specimen, biopsy is invasive and associated with inherent risks such as bleeding and infection as well as the potential for metastasis [1], [2]. These risks are a matter of serious concern considering that most adrenal tumors, especially those found incidentally, are benign [1], [2], [6]. A study by Kaperlik-Zaluska et al. [7] reported that of 1,444 incidentally discovered adrenal tumors, 87% were considered benign.

Radiographic methods of differentiating benign and malignant tumors may address these concerns, and help avoid unnecessary procedures as well as improve outcomes. Computed tomography (CT) perfusion imaging has been used to differentiate adrenal adenomas from non-adenomas by observing the time density curve, four CT perfusion parameters (blood flow [BF], blood volume [BV], mean transit time [MTT], and permeability surface area product [PS]), and perfusion images. Our previous results indicated that a BV≥9.325 ml/min/100 g had a sensitivity and specificity of 76.9% and 73.2%, respectively, for the diagnosis of adrenal adenomas [8].

Studies have demonstrated that tumor angiogenesis is closely related to biological behavior, degree of differentiation, and tumor prognosis [9]–[11]. Currently, the detection of vascular endothelial growth factor (VEGF) expression and microvessel density (MVD) by immunohistochemistry is the gold standard in the evaluation of tumor angiogenesis. However, immunohistochemistry staining for MVD and VEGF requires an invasive biopsy of the tumor. Investigating the correlations between CT perfusion parameters and expression of MVD and VEGF in adrenal tumors may allow us to further understand the value of CT perfusion parameters in monitoring angiogenesis of adrenal tumors, and non-invasively determining their clinical behavior.

Thus, the aim of this study was to measure CT perfusion parameters and their correlation with MVD and VEGF in adrenal adenomas with non-adenomas to determine if perfusion CT can be used to distinguish adenomas from non-adenomas.

## Materials and Methods

### Subjects

This study was carried out between October 2004 and February 2006 with a total of 34 patients with adrenal tumors. The Institutional Review Board of the General Hospital of Tianjin Medical University approved this study, and written consents were obtained from all patients before initiation of the study. Histopathological diagnosis of the lesions was available for all subjects. Subjects were included if they: (1) had a tentative diagnosis of an adrenal gland tumor; (2) had an incidental diagnosis of adrenal gland mass on chest or abdominal CT examination; (3) had a primary tumor suspected to have adrenal gland metastases; (4) did not undergo surgery initially, but the lesion was diagnosed by laboratory testing (e.g., Conn's adenoma); and (5) had surgical resection for pathological confirmation of the diagnosis. Subjects were excluded if: (1) CT examination revealed no adrenal gland tumor; (2) they failed to complete CT perfusion examination; (3) their CT perfusion curve could not be generated or perfusion parameters could not be measured due to respiratory motion artifacts resulting in loss of the selected tumor scanning slice; (4) they did not undergo surgery during follow-up or laboratory indexes could not identify the characteristics of their tumor; (5) the specimens obtained were not properly stored such that pathological examination could not be performed.

### Perfusion CT imaging

CT scanning was performed with Lightspeed Pro 16-slice spiral CT (GE Medical Systems, Milwaukee, Wis., USA). Patients received respiratory training in order to hold their breath as long as possible to reduce respiratory artifacts.

Conventional plain CT scanning was performed to localize lesions. Patients were placed in the supine position and a non-enhanced CT scan was performed from the T1 to L2 vertebral levels. Conventional plain CT scanning parameters for 16-slice spiral CT were 120 kVp, 230 mA, collimator width 0.625 mm, reconstruction slice thickness 2.5–5 mm, pitch 1.375, matrix size 512×512, field of view (FOV) 20–22 cm, and 1 slice/s.

In conventional plain CT scanning, the slice with the maximum tumor cross-sectional area or the central slice with minimum visible necrosis, cystic lesions, hemorrhage, or calcifications was selected for subsequent perfusion CT scanning. A high-pressure injector (Medrad® MCT/MCT Plus CT Stellant®, Pittsburgh, Penn, USA) was used to intravenously inject non-ionic iodinated contrast medium (Ultravist [370 mg/ml]; Ansheng Pharmaceutical Company, Shanghai, China) through the antecubital vein at a speed of 4.5 ml/s with a total volume of 50 ml. Scanning was performed at the target plane beginning 7–9 s after injection. After contrast injection, 20–40 ml of normal saline was intravenously administered at the same speed.

The perfusion CT imaging was performed using a CINE scan mode. For 16-slice spiral perfusion CT scanning, perfusion scanning was performed at 2 cm in the target plane, and the parameters were 80 kVp, 100 mA, collimator width 0.625 mm, reconstruction slice thickness 2.5–5 mm, pitch 1.375, matrix size 512×512, FOV 20–22 cm, and 4 or 8 slices/s. Scanning was performed for 40 s, following the manufacturer's instructions. Thus, 320 or 160 images (40 images ×8 or 40 images ×4) were obtained in a 16-slice perfusion CT scan. The reconstruction slice thickness was dependent on the tumor size, i.e., the size of the adrenal tumors ranged from <1.0 cm to >10 cm.

### Image processing, observations, and parameters for measurement

Details of the imaging processing and measurements have been previously described [8]. Briefly, image processing was performed on an Image Advantage CT workstation with version 4.2 software (AW4.2 Medical Systems, Milwaukee, Wis., USA). The abdominal aorta was selected as the input artery. When the appropriate region of interest (ROI) was placed, perfusion software automatically generated the time density curve (TDC) of the abdominal aorta and adrenal tumors. At the same time, parametric map images were obtained of the four perfusion parameters reflecting tumor perfusion status (BF, BV, MTT, and PS). BF was defined as the blood volume flowing across 100 g of tumor per min (ml/min/100 g); BV was defined as the total blood volume flowing across 100 g of tissue (ml/100 g); MTT was defined as mean time taken for blood to flow from the artery to the vein (s); and PS was defined as the product of the capillary endothelial space and the sum of their surface area, which reflects the blood volume flowing from the capillaries to the interstitial space (ml/min/100 g).

Areas of visible necrosis, cystic areas, and areas of hemorrhage and calcification were excluded from the ROIs. The ROI was drawn to specifically avoid perfused regions consistent with the aortic perfusion curve in order to exclude the influence of non-tumoral blood vessels and thus minimize possible partial volume effects; the size of the ROI was >20 mm^2^, and the minimum area was 4–16 mm^2^ [12]. The ROI selected was of a homogenous texture, was usually round or oval in shape, and its area was 1/2 to 2/3 of the tumor cross-sectional area. In addition, for tumors with uneven texture, at least three ROIs were placed in the solid part of the maximal cross-sectional area and/or on different slices. ROI perfusion values were then recorded, and the average value was calculated. Afterwards, the adrenal tumor BF, BV, MTT and PS were recorded.

### Pathological examination

Tumor samples were prepared for pathological examination from the same approximate location and in the same orientation as the analyzed CT images. Visual inspection of gross tumor tissue was compared with CT images to ensure maximal consistency of the areas subjected to pathological examination with the ROIs selected on the CT images. Tissue specimens were fixed in 10% neutral formaldehyde, and the formaldehyde-fixed tissues were then embedded in paraffin. As the reconstruction slice thickness of CT was 2.5–5 mm, specimens were cut into 4-µm sections with the intention to obtain the closest match between the CT ROI and tumor specimens examined. Three consecutive sections were collected from each tumor for hematoxylin and eosin (HE) staining and immunohistochemistry (VEGF and MVD); different ROIs were obtained from different CT slices, thus the three consecutive sections came from each matched but thicker CT slice.

Two-step immunohistochemistry was performed using the immunoglobulin-peroxidase method. Mouse anti-human VEGF monoclonal antibody, mouse anti-human CD34 monoclonal antibody, goat anti-mouse PV-6000 antibody, and DAB kit were purchased from Beijing Zhongshan Biotech Co., Ltd, Beijing, People's Republic of China. The immunohistochemistry images were acquired by the CMIAS multifunctional image analysis system and AGFA-II scanner (Image Center of Beijing University of Aeronautics & Astronautics, Beijing, People's Republic of China) and the MVD and VEGF expression were determined. Cells staining positive for endothelial cell marker CD34 had brown or yellow-brown granules, and microvessels with positive-staining cells without background were marked and counted to determine the MVD. According to the criteria developed by Weidner et al. [13], the microvessels with identifiable positive-staining cells or cell clusters served as a blood vessel. Sections were then observed at low magnification (×40), and then three regions with high blood vessel density were observed at a higher magnification (×200; 0.739 mm^2^), photographed, and then the average density of blood vessels was calculated for the three regions. VEGF-positive cells were recognized by brown or yellow-brown granules in the cytoplasm or on the cell membrane, a light blue nucleus, and absent background. According to the classification criteria developed by Liu et al. [14], the VEGF expression was classified into four grades: grade I, <25%; grade II, 25∼50%; grade III, 50∼75%; grade IV, >75%.

### Statistical analysis

Testing for normality was performed for continuous data with the Shapiro-Wilk test. When both groups (adrenal adenomas and non-adenomas) had normally distributed data, mean and standard deviation (SD) were presented, and comparisons were made by the independent t-test. When either of the groups had non-normally distributed data, median and inter-quartile range (IQR; range between the 25^th^ and 75^th^ percentile) were presented and the comparison was conducted with the Mann-Whitney U test. Categorical data were presented by frequency (percentage) and compared by the Chi-square test. The correlation between VEGF, MVD, and CT perfusion parameters (BF, BV, MTT, PS) was measured by Spearman's rank correlation coefficient for total patients, adenomas, and non-adenomas, denoted as r_total_, r_adenoma_, and r_non-adenoma_, respectively. The correlation strength was compared with the following scale: very weak (0–0.19), weak (0.20–0.39), moderate (0.40–0.59), strong (0.60–0.79), and very strong (0.80–1.00). The statistical analyses were performed with SAS software version 9.2 (SAS Institute Inc., Cary, North Carolina, USA) using a two-tailed *p*<0.05 to indicate statistical significance.

## Results

### Patients

There were a total of 45 patients who met the inclusion criteria, and of these 11 were excluded. The cases that were excluded included four cysts, four adenomas, one myelinoma, one hematoma, and one case in which the pathological features were indeterminate. The causes for exclusion included failure of perfusion because respiratory movement caused the split-level phenomenon, measurement of perfusion was infeasible in the scanning area, the samples were not suitable for pathological examination due to poor preservation, patients were not suitable for surgical intervention, and pathological features could not support a diagnosis. Thus, there were 16 males and 18 females with a mean age of 43±11 years (range: 23 to 64 years) included in the study. Of the 34 patients, 18 had tumors found in the left adrenal gland and 16 in the right adrenal gland; one patient had bilateral adrenal tumors. Patient data is summarized in Table 1. The tumor sizes ranged from 0.8×1.0 cm to 11.5×10.0 cm (mean: 3.0±1.8 cm ×2.8±1.4 cm). According to the initial diagnosis prior to inclusion in the study, the tumors included 17 adrenal adenomas (50%) and 17 adrenal non-adenomas (50%). The adrenal adenomas were comprised of 10 aldosterone-producing tumors (29%), five non-functioning tumors (15%), and two cortisol-producing tumors (6%). The adrenal non-adenomas were comprised of 13 pheochromocytomas (38%), two adrenocortical carcinomas (6%), one adrenal myelolipoma (3%), and one ganglioneuroma (3%).

**Table 1:** Patient data.

Characteristics	N = 34
Age (y)	43±11
Gender	
Male	16 (47)
Female	18 (53)
**Adrenal adenomas**	**17 (50)**
Conn's	10 (29)
Non-functioning	5 (15)
Cushing's	2 (6)
**Adrenal non-adenomas**	**17 (50)**
Pheochromocytoma	13 (38)
Adrenocortical carcinoma	2 (6)
Adrenal myelolipoma	1 (3)
Ganglioneuroma	1 (3)

Data are presented as mean ± SD or number (%).

### Comparison of perfusion CT parameters between adenomas and non-adenomas

Perfusion CT imaging was successful in all 34 patients. A comparison of the four CT perfusion parameter values (BF, BV, MTT, and PS) between adenomas and non-adenomas is shown in Table 2. The median BV was significantly higher in adenomas than in non-adenomas [12.3 (10.4, 16.5) ml/100 g vs. 8.8 (3.3, 9.4) ml/100 g, *p* = 0.001; Mann-Whitney U test]. However, differences in BF, MTT, and PS parameter values between adenomas and non-adenomas were not significant (all, *p*>0.05).

**Table 2:** Comparison of CT perfusion parameters between adrenal adenomas and non-adenomas.

CT perfusion parameter	Adrenal adenomas (*n* = 17)	Adrenal non-adenomas (*n* = 17)	*p*
BF (ml/min/100 g)^2^	93 (76–123)	102 (29–148)	0.5†
BV (ml/100 g)^2^	12.3 (10.4–16.5)	8.8 (3.3–9.4)	0.001† *
MTT (s)^1^	11±3	11±5	0.9‡
PS (ml/min/100 g)^1^	28±18	17±15	0.07‡

Data are presented as mean±SD^1^ or median (IQR)^2^.

† Mann-Whitney U test; ‡independent t-test.

* Statistically significant, *p*<0.05.

### Comparison of MVD and VEGF between adenomas and non-adenomas

A comparison of MVD and VEGF in adenomas and non-adenomas is shown in Table 3. The mean MVD was significantly higher in adenomas as compared to non-adenomas (98±28 vs. 53±27, *p*<0.0001; independent t-test). Adenomas also expressed significantly more VEGF than non-adenomas [65 (50, 79) % vs. 45 (35, 67) %, *p* = 0.02; Mann-Whitney U test]. Twelve adenoma cases (71%) had high VEGF expression (III+IV), but only five non-adenoma cases (29%) showed similar high levels, resulting in a significant difference in high VEGF expression between adenomas and non-ademonas (*p* = 0.02; Chi-square test).

**Table 3:** Comparison of MVD and VEGF between adrenal adenomas and non-adenomas.

	Adrenal adenomas (*n* = 17)	Adrenal non-adenomas (*n* = 17)	*p*
MVD^1^	98±28	53±27	<0.0001‡
Median VEGF expression (%)^2^	65 (50, 79)	45 (35, 67)	0.02† *
VEGF grade (%)^3^			0.02¶*
I+II	5 (29)	12 (71)	
III+IV	12 (71)	5 (29)	

Data are presented as mean±SD^1^ or median (IQR)^2^ for continuous variables or number (%)^3^ for categorical variables.

† Mann-Whitney U test; ‡independent t-test; ¶Chi-square test.

* Statistically significant, *p*<0.05.

### Correlation between MVD and VEGF

**Figure 1:**
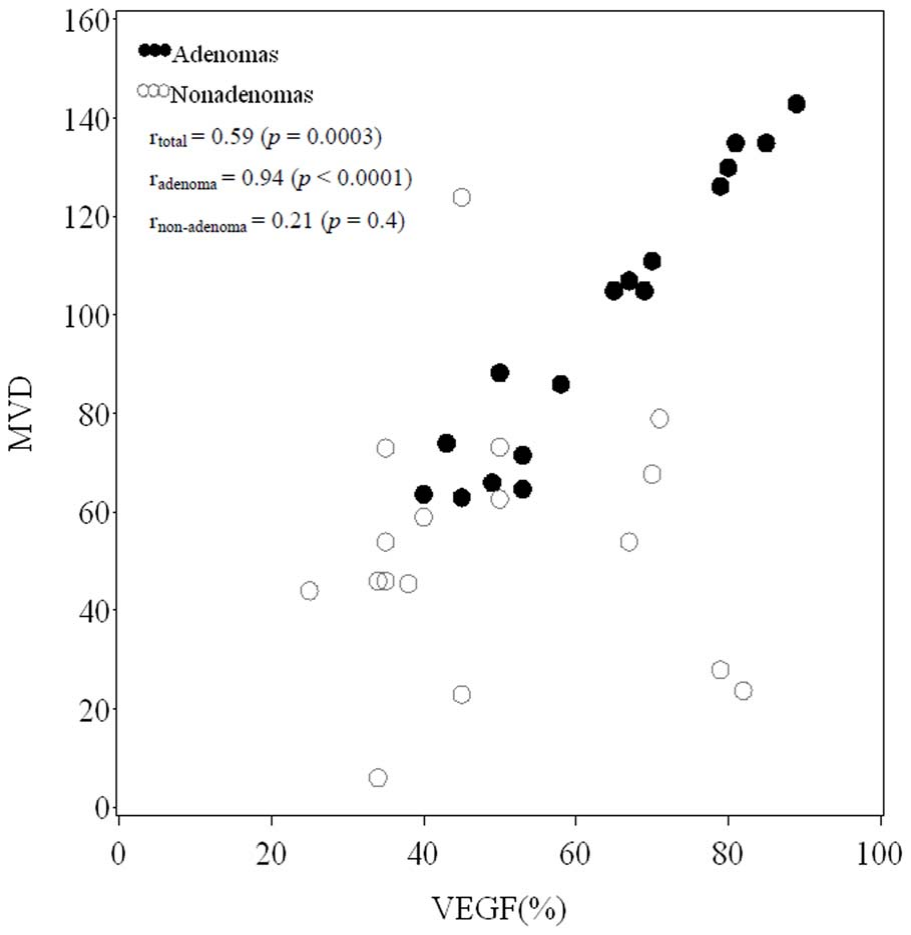
Scatter plot of MVD versus VEGF. Spearman's rank correlation coefficients for total patients, adenomas, and non-adenomas were r_total_ = 0.59 (*p* = 0.0003), r_adenoma_ = 0.94 (*p*<0.0001), and r_non-adenoma_ = 0.21 (*p* = 0.4), respectively.

A moderately positive correlation between MVD and VEGF for all patients, including those with adenomas and non-ademonas, was found and demonstrated in Figure 1 (r_total_ = 0.59, *p* = 0.0003). Figure 1 also demonstrates the correlation between MVD and VEGF was very strong for adenomas (r_adenoma_ = 0.94, *p*<0.0001), but weak for non-adenomas (r_non-adenoma_ = 0.21, *p* = 0.4).

### Correlation between CT perfusion parameters and VEGF and MVD

**Figure 2:**
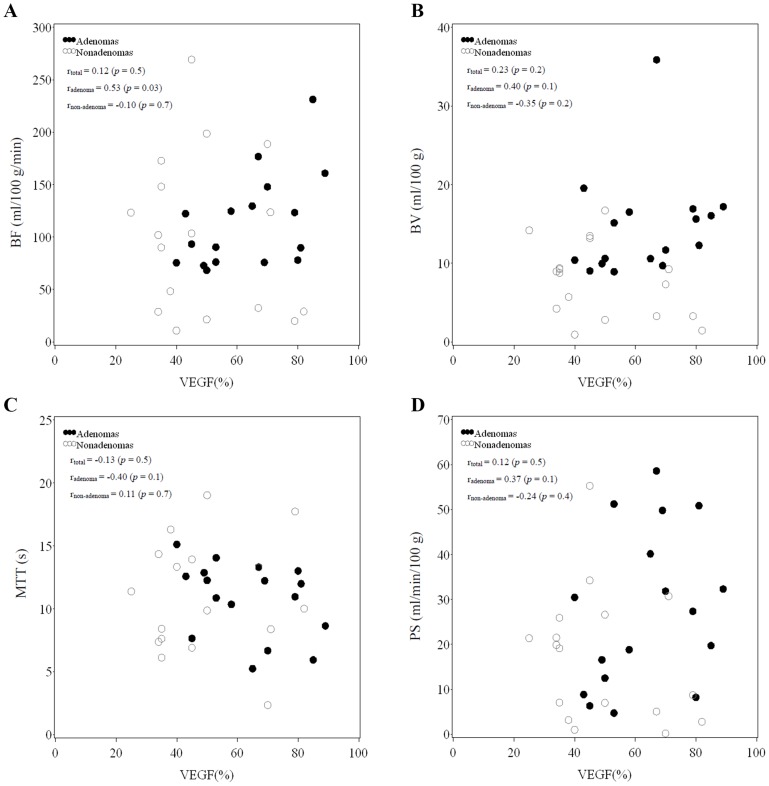
Scatter plots of CT perfusion parameters (BF, BV, MTT, PS) versus VEGF. Spearman's rank correlation coefficients for total patients, adenomas, and non-adenomas, denoted by r_total_, r_adenoma_, and r_non-adenoma_, respectively.

**Figure 3:**
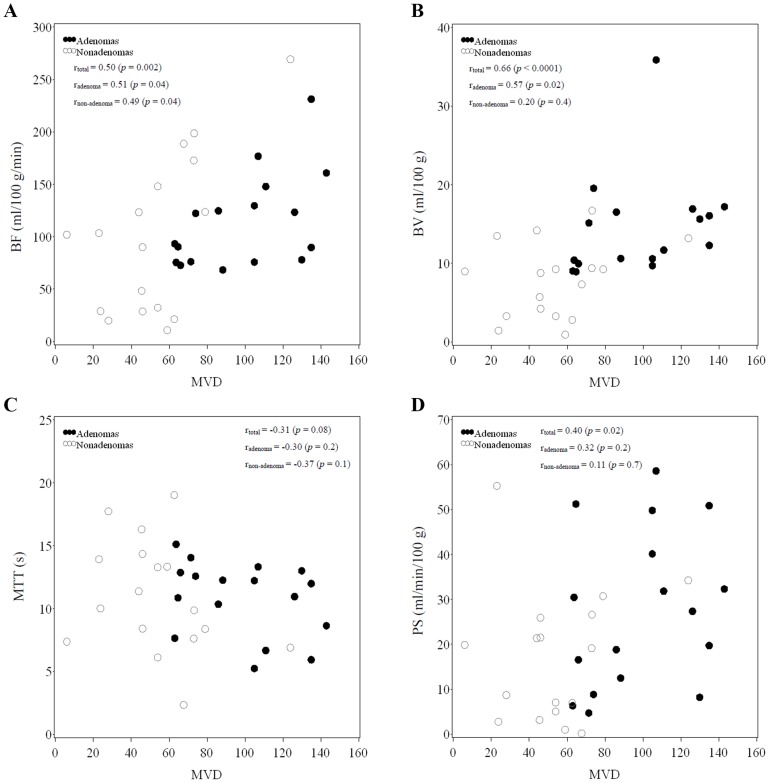
Scatter plots of CT perfusion parameters (BF, BV, MTT, PS) versus MVD. Spearman's rank correlation coefficients for total patients, adenomas, and non-adenomas, denoted by r_total_, r_adenoma_, and r_non-adenoma_, respectively.

Four scatter plots illustrated the relationships between VEGF vs. CT perfusion parameters BF (Figure 2A), BV (Figure 2B), MTT (Figure 2C), and PS (Figure 2D). Scatter plots were also used to illustrate the relationships between MVD vs. CT perfusion parameters BF (Figure 3A), BV (Figure 3B), MTT (Figure 3C) and PS (Figure 3D). Correlation data for Figure 2 and 3 are summarized in Table 4. Among the total number of patients (adenomas + non-adenomas), there was no significant correlation found between the four CT perfusion parameters and VEGF (all, *p*>0.05). However, there was a moderately strong correlation between BF and VEGF among adenomas (r_adenoma_ = 0.53, *p* = 0.03). Among the total number of patients (adenomas + non-adenomas), significantly positive correlations were observed between BF and MVD (r_total_ = 0.50, *p* = 0.002), BV and MVD (r_total_ = 0.66, *p*<0.0001), and PS and MVD (r_total_ = 0.40, *p* = 0.02). However, there was a weakly negative but non-significant correlation observed between MTT and MVD, (r_total_ = −0.31, *p* = 0.08). Although the correlation between BF, BV, and MVD were significant for both adenomas and non-adenomas (adenomas: BF: r_adenoma_ = 0.51, *p* = 0.04; r_non-adenoma_ = 0.49, *p* = 0.04; BV: r_adenoma_ = 0.57, *p* = 0.02; r_non-adenoma_ = 0.20, *p* = 0.02), the difference in correlation between BF and MVD in adenomas vs. non-adenomas was minor.

**Table 4:** CT perfusion parameter correlation with VEGF and MV.

	VEGF (%)			MVD		
CT perfusion parameter	r_total_	r_adenoma_	r_non-adenoma_	r_total_	r_adenoma_	r_non-adenoma_
BF (ml/min/100 g)	0.12	0.53^*^	−0.10	0.50^**^	0.51^*^	0.49^*^
BV (ml/100 g)	0.23	0.40	−0.35	0.66^***^	0.57^*^	0.20
MTT (s)	−0.13	−0.40	0.11	−0.31	−0.30	−0.37
PS (ml/min/100 g)	0.12	0.37	−0.24	0.40^*^	0.32	0.11

Spearman's rank correlation coefficient for total patients, adenomas, and non-adenomas, denoted as r_total_, r_adenoma_, and r_non-adenoma_, respectively.

Statistically significant: ^*^ *p*<0.05; ^**^ *p*<0.01; ^***^ *p*<0.001.

### Structural characteristics of blood vessels, MVD and VEGF staining of adenomas and non-adenomas

**Figure 4:**
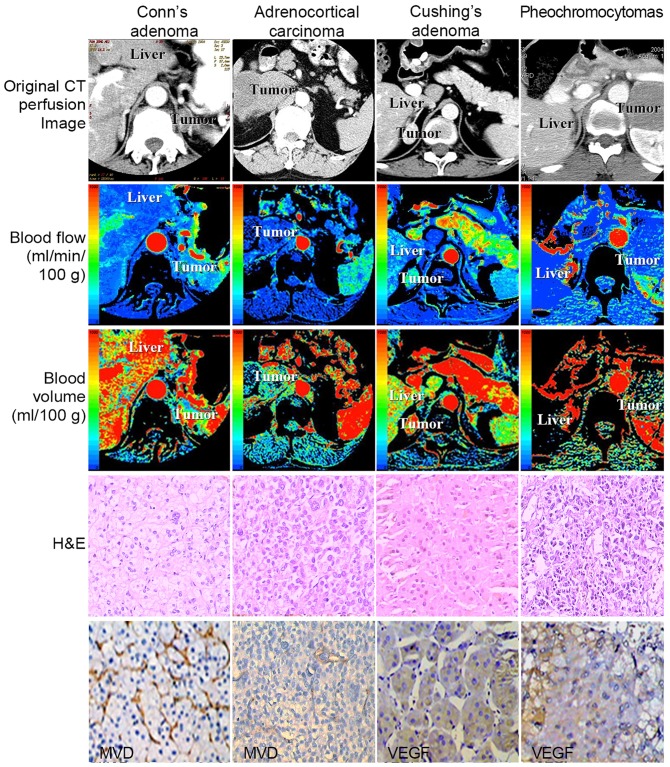
Representative CT perfusion images, BF, BV, hematoxylin and eosin staining, and MVD or VEGF immunostaining of aldosterone-producing adenoma (Conn's), adrenocortical carcinoma, cortisol-producing adenoma (Cushing's), and pheochromocytoma.

Three histological sections were analyzed for each of the 34 tumors, for a total of 102 histological sections. The analysis was performed by a team of three pathologists. Representative images of adenomas and non-adenomas are presented in Figure 4. Conn's adenomas were rich in microvessels that were evenly distributed, with long or short-cord morphology, running in a straight course, and exhibiting simple branches. The distribution was similar throughout the tumor, i.e., the same in the center and periphery. For non-adenomas (adrenocortical carcinoma), the blood vessels were unevenly distributed and lumen structures were observed in MVD staining. VEGF protein was evenly and highly expressed in adenoma tissue (Cushing's adenoma), while non-adenomas (pheochromocytomas) expressed a lower level of VEGF protein in an uneven distribution, especially at the edge of the tumor tissue and areas around large blood vessels.

## Discussion

The results of this study showed that VEGF was evenly and highly expressed in adenoma tissue, while non-adenomas expressed a lower level of VEGF protein in an uneven distribution, and the mean MVD was significantly higher in adenomas as compared to non-adenomas. A strong positive correlation between MVD and VEGF expression was present in adenomas. In adenomas there was positive correlation between BV, MVD and VEGF expression. These results suggest that the perfusion CT parameter BV may be useful for distinguishing adenomas from non-adenomas.

### ROI selection

Severe necrosis in a tumor may influence the determination of ROI and its measured perfusion data. Ideally, differentiation between well-perfused and poorly-perfused areas of a tumor in CT would allow for selection of an ROI with homogeneous perfusion. However, in reality the texture of the tumor influences the perfusion pattern: when the tumor has a homogenous texture, it is hard to differentiate well-perfused vs. poorly-perfused areas; when the tumor has greater necrosis, there is also no clear boundary between well-perfused vs. poorly-perfused areas. In most tumors with greater necrosis identified histologically, the blood vessels are frequently richly distributed in the solid part at the periphery of tumor, the necrotic part possesses no blood supply, and there is no clear-cut border between the necrotic region and the solid peripheral region. Areas with inadequate blood supply with fluctuating levels of perfusion, red blood cell flux, and hypoxia, as well as abnormal metabolic activity have been well documented [15]–[18]. Since MVD is inconsistent in adenomas vs. non-adenomas [19], we propose that during the measurement of adrenal tumor perfusion reference to the corresponding CT images to identify areas of visible necrosis, cystic areas, and areas of hemorrhage and calcification are important for the selection of the ROI.

### Microvessel density and characteristics

Our results showed that the mean MVD was significantly higher in adenomas as compared to non-adenomas (Table 3). In our study, the most common non-adenoma was the benign chromaffinoma, and some chromaffinomas had relatively high MVD (the highest MVD was 124). Although two adrenocortical carcinomas were malignant tumors, their MVD was low. However, in the present study, the two cortisol-producing adenomas had the highest MVD followed by the aldosterone-producing adenoma; the lowest MVD was found in the non-functional adenoma. A study of adrenocortical tumors and normal adrenal cortex angiogenesis by Bernini et al. [19] in which vessel density was calculated based on CD34 positive staining showed that MVD was the highest in normal adrenal cortex while lower in aldosterone-producing adenomas, cortisol-producing adenomas, and non-functional adenomas, with the lowest MVD seen in adrenocortical carcinoma. The reason our results differ from those of Bernini et al. [19] is possibly the small sample size.

In this study, we observed that the general characteristics of microvessels in adenomas were simple branching, long or short cord-like morphology, straighter course, and mostly invisible lumen. Among non-adenomas, pheochromocytoma demonstrated microvascular lumen sinusoid dilation, reticular branching structures, and an irregular course, and ganglioneuroma and adrenocortical carcinoma demonstrated sparse microvessels distribution (Table 5). We also observed widened and regular microvessels in two patients with Cushing's tumors. In the two patients with cortical carcinoma, the distribution of microvessels was different: in one patient the microvessels were rich in the periphery of tumor, had enlarged microvascular lumens, and were irregular, while in the other patient microvessels were rare but the tumor was rich in tumor cells. On the basis of these findings, we speculate that the pattern of microvessels varies within adenomas and within non-adenomas (as well as between adenomas and non-adenomas).

**Table 5:** General microvessel characteristics of adenomas and non-adenomas.

Adenomas	Non-adenomas		
	Adrenocortical carcinoma	Pheochromocytoma	Ganglioneuroma
Simple branching, long or short cord-like morphology, straighter course, and mostly invisible lumen	Sparse microvessel distribution	Microvascular lumen sinusoid dilation, reticular branching structures, and an irregular course	Sparse microvessel distribution

### VEGF expression

Our results showed that VEGF is expressed within adrenal tumor cells, and the level of expression varies (Table 3). Some tumors demonstrated stronger expression at the tumor periphery and surrounding the larger vessels, and VEGF expression was higher in adenomas than non-adenomas. In particular, overexpression was noted in cortical carcinoma, and lower expression was noted in Conn's adenoma and Cushing's adenoma, which is consistent with a previous report by Bernini et al. [19]. Britvin et al. [20] found that serum VEGF levels were significantly higher in patients with adrenal tumors than in healthy controls, and that levels were greater in patients with adrenocortical cancer. Similarly, Kolomecki et al. [21] reported that serum VEGF levels were higher in patients with adrenal tumors than healthy controls and the levels were significantly higher in patients with malignant than benign tumors.

In general, our findings are consistent with those of Yancopoulos et al. [22] who showed that VEGF expression in tumors is closely related to tumor angiogenesis, with higher VEGF expression corresponding to higher MVD, and vice versa. However, there exist exceptions where the relationship may not always be a positive correlation. For instance, in the present study, two adrenocortical carcinomas had grade III and grade IV levels of VEGF expression, but there was relatively low MVD in both tumors, which was in accordance with other reports [8], [23]. In these 2 cases, extensive necrosis was observed, MVD was at a relatively low level, and thus VEGF expression was not proportional to MVD. We speculate that (1) other angiogenesis inhibitory factors may have antagonized the functional activity or angiogenic activity of VEGF, or (2) in adrenal cortical carcinoma VEGF might not be a major factor inducing angiogenesis [19].

### Correlations between MVD, VEGF, and perfusion parameters

The correlation between MVD and VEGF was much stronger for adenomas than for non-adenomas (Table 4, Figure 1). Adenomas originate from the adrenal cortex, with some of them possessing secretory functions, and despite their benign nature they possess rich vascularity due to functional requirements. In a study of adrenal cortical tumors and adrenal cortical angiogenesis, Bernini et al. [19] reported that Conn's adenoma possesses a very similar MVD as compared to normal adrenal cortex, with Cushing adenoma as the next most similar, followed by non-functional adenomas. In addition, MVD was positively correlated with aldosterone in Conn's adenomas, but not correlated with steroids and ACTH. This indicated that there exists a certain association between an adrenal cortical tumor and its functional status. Conn's adenoma also had a higher level of VEGF expression than normal adrenal tissue, Cushing adenomas, and non-functional adenomas. Thus, the stronger correlation of MVD and VEGF expression in adenomas as compared to non-adenomas in this study is consistent with existing literature.

In our study, both VEGF expression and MVD of adenomas were positively correlated to BV, BF and PS but negatively correlated with MTT, with the strongest correlations noted between BV and BF with VEGF expression and MVD, as well as between VEGF expression and MVD (Table 4, Figures 2, 3). While in non-adenomas, although MVD was positively correlated with BV and BF, correlation between VEGF expression and MVD was weak. Based on both adenoma and non-adenoma CT perfusion scan findings and angiogenesis detection, the levels of BF, BV, MVD and VEGF expression are not always consistent with one another. Under pathological conditions, VEGF-mediated angiogenesis may be different than that occurring in a normal physiological environment; specifically, there is a difference in the structure and morphology of tumor blood vessels. Both affect tumor MTT and PS, and thus VEGF expression is still related to MTT and PS.

There are some limitations to this study that should be considered. First, the subject sample size is relatively small. Furthermore, the group of participants was from a single institution and therefore the representation of the subjects is far from being a comprehensive reflection of tumor pathology. In addition, the population consisted of patients in whom the tumors were resected, which may have led to a bias. Placement of the ROI involved radiologists' determination and variability could not be avoided. In addition, the pattern of perfusion in tumors may be averaged out with the thickest CT slices, and the difference between the sizes of individual adrenal tumors in this study was large, which may bias the results. Lastly, we did not measure muscle perfusion as an internal reference perfusion value; determining tumor perfusion relative to muscle perfusion may have increased the accuracy of the results.

## Conclusions

In summary, morphology, MVD, and VEGF expression differ significantly between adenomas and non-adenomas. Of the CT perfusion parameters examined, both BF and BV correlate with MVD, but only BF correlates with VEGF, and only in adenomas. The significant difference in BV suggests that this parameter may be used to differentiate adenomas from non-adenomas. However, the small size of difference in BV shows that it may only be possible to use this parameter to identify adenomas vs. non-adenomas at extreme BV values. Examination of much larger clinical datasets would be required to ascertain the value of BV in this context.
